# *De Novo* Transcriptome Assembly of the Chinese Swamp Buffalo by RNA Sequencing and SSR Marker Discovery

**DOI:** 10.1371/journal.pone.0147132

**Published:** 2016-01-14

**Authors:** Tingxian Deng, Chunying Pang, Xingrong Lu, Peng Zhu, Anqin Duan, Zhengzhun Tan, Jian Huang, Hui Li, Mingtan Chen, Xianwei Liang

**Affiliations:** Key Laboratory of Buffalo Genetics, Breeding and Reproduction technology, Ministry of Agriculture, Buffalo Research Institute, Chinese Academy of Agricultural Sciences, Nanning, Guangxi, P. R. China; Kunming University of Science and Technology, CHINA

## Abstract

The Chinese swamp buffalo (*Bubalis bubalis*) is vital to the lives of small farmers and has tremendous economic importance. However, a lack of genomic information has hampered research on augmenting marker assisted breeding programs in this species. Thus, a high-throughput transcriptomic sequencing of *B*. *bubalis* was conducted to generate transcriptomic sequence dataset for gene discovery and molecular marker development. Illumina paired-end sequencing generated a total of 54,109,173 raw reads. After trimming, *de novo* assembly was performed, which yielded 86,017 unigenes, with an average length of 972.41 bp, an N50 of 1,505 bp, and an average GC content of 49.92%. A total of 62,337 unigenes were successfully annotated. Among the annotated unigenes, 27,025 (43.35%) and 23,232 (37.27%) unigenes showed significant similarity to known proteins in NCBI non-redundant protein and Swiss-Prot databases (E-value < 1.0E-5), respectively. Of these annotated unigenes, 14,439 and 15,813 unigenes were assigned to the Gene Ontology (GO) categories and EuKaryotic Ortholog Group (KOG) cluster, respectively. In addition, a total of 14,167 unigenes were assigned to 331 Kyoto Encyclopedia of Genes and Genomes (KEGG) pathways. Furthermore, 17,401 simple sequence repeats (SSRs) were identified as potential molecular markers. One hundred and fifteen primer pairs were randomly selected for amplification to detect polymorphisms. The results revealed that 110 primer pairs (95.65%) yielded PCR amplicons and 69 primer pairs (60.00%) presented polymorphisms in 35 individual buffaloes. A phylogenetic analysis showed that the five swamp buffalo populations were clustered together, whereas two river buffalo breeds clustered separately. In the present study, the Illumina RNA-seq technology was utilized to perform transcriptome analysis and SSR marker discovery in the swamp buffalo without using a reference genome. Our findings will enrich the current SSR markers resources and help spearhead molecular genetic research studies on the swamp buffalo.

## Introduction

The water buffalo (*Bubalus bubalis*), which belongs to the *Bubalus* genus of the *Bovidae* family, is an economically significant livestock that has been used as dairy, meat, and source of draught power [1]. These animals are typically found in tropical and subtropical regions, wet grasslands, marshes and swamps. The domestic water buffalo in Asia is generally classified into two major subspecies based on body size, outward appearance, biological characteristics, and chromosome karyotype, namely, the river buffalo (2n = 50) and the swamp buffalo (2n = 48) [2]. In China, the native buffaloes are of the swamp type, and are mainly distributed in 18 provinces of central and southern China, and have been divided into 18 local breeds based on regional distribution [3, 4]. In the past, swamp buffaloes were mainly raised by small-scale farmers for draught power for agricultural production. However, considering its economic importance as the provider of milk, meat, horns and even skin, extensive efforts on the genetic improvement of the dairy buffalo were conducted for several decades in China using a crossbreeding system. The milk yield of crossbreeds Murrah F1 and F2 reached 1,240.5 kg and 1,423.3 kg respectively, which were 13.5% and 30.2% higher than that of selected local buffaloes (*P* < 0.01). The milk yield of crossbreeds Nili-Ravi F1 and F2 reached 2,041.2 kg and 2,351.3 kg respectively, which were 86.8% and 115.2% higher than that of selected buffaloes (*P* < 0.01) [5]. Although milk yield performance has markedly improved in crossbreeds compared to indigenous buffaloes, the average milk yield per lactation of crossbreeds is still far lower than that of purebred Murrah, Nili-Ravi, and Mediterranean buffaloes [6, 7]. One of the main long-term hindrances in the buffalo industry in China is the lack of breeds with high milk and reproductive performance.

With the purpose of increasing the size of the dairy buffalo herd and improving the production performance of dairy buffalo, previous studies have mainly focused on reproductive technologies [8, 9], the identification of genes and molecular markers that were associated to desirable traits [10–12], genetic relationships, and genetic variations [13, 14]. To date, information on the technology for buffalo genetic breeding in China is limited, particularly relating to molecular breeding methods. One key impediment is lack of genomic information on the buffalo, which could be utilized in development of molecular markers for its selection and breeding. Several research groups have conducted genomic studies on the buffalo [15, 16], which has recently resulted in the release of the draft genome of the river buffalo [17], and is expected to play an important role in promoting the genetic improvement of the dairy buffalo. However, no published genome sequence is currently available for the swamp buffalo, which in turn may hinder molecular genetic studies on buffalo breeding.

Transcriptome studies have become an important method to obtain large amounts of sequence data that could enrich the genome resource for the non-model animals [18]. RNA sequencing (RNA-seq) is a high throughput technology that has been effectively utilized in transcriptional analysis, gene discovery, and development of molecular markers in various species such as human [19], cattle [20], sheep [21], goat [22] and pig [23]. The genetic relationship and diversity among different buffalo breeds have been mainly investigated using restriction fragment length polymorphism (RFLP) [24], random amplified polymorphic DNA (RAPD) [25], single nucleotide polymorphism (SNP) [26], and simple sequence repeat (SSR) [27] markers. SSR markers have been demonstrated to be an extremely useful tool for investigating population clustering, genetic divergence, parentage testing, and genetic resource conservation[28–30]. Sarika et al. [31] developed the first microsatellite database of the water buffalo, *BuffSatDb* (http://cabindb.iasri.res.in/buffsatdb/), which is a web-based relational database of 910,529 microsatellite markers that was generated by *in silico* microsatellite mining and has helped in resolving the presence of degenerate bases in the current buffalo assembly. However, SSR markers that have been used in the analysis of genetic relationships and genetic variations of different buffaloes were mainly derived from other domesticated bovids [32–34], and no SSR markers of the swamp buffalo have been developed and reported to date. Specifically, suitable SSR markers that could be used to improve the production performance of dairy buffalo are very scarce, and have yet to be developed. Therefore, a large-scale and low-cost approach is required to develop SSR markers for the swamp buffalo. In the present study, we performed Illumina paired-end sequencing of pooled tissues of the swamp buffalo to generate a set of unigenes that were used to develop SSR markers. Then, we identified novel SSR markers in the swamp buffalo which can be utilized for marker identification, parentage testing, genetic resource conservation, and molecular breeding.

## Materials and Methods

### 1. Ethics statement

All animal procedures and study design were conducted in accordance with the Guide for the Care and Use of Laboratory Animals (Ministry of Science and Technology of China, 2006) and were approved by the Animal Ethics Committee of the Buffalo Research Institute, Chinese Academy of Agricultural Sciences.

### 2. Animal materials and RNA extraction

Two swamp buffaloes (male and female) were obtained from the Buffalo Research Institute, Chinese Academy of Agricultural Sciences (Nanning, China) and slaughtered by exsanguination. Fresh tissue samples were collected, including the heart, brain, lung, kidney, fat, liver, spleen, uterus, testis, ovary, and gland, immediately frozen in liquid nitrogen and stored at -80°C until use.

Total RNA was extracted from each collected tissues sample using the TRIzol reagent following the manufacturer’s specifications (Invitrogen, Guangzhou, China). The quality and quantity of each RNA sample was measured by using an Agilent 2100 Bioanalyzer (Agilent Technologies, Santa Clara, CA, USA). A total of 16.5 μg of RNA was equally pooled from the collected tissues for cDNA library preparation.

### 3. Transcriptome sequencing

The mRNAs were isolated from total RNA using a Dynabeads mRNA DIRECT Kit (Invitrogen, Guangzhou, China) and fragmented into short fragments with a fragmentation buffer. By using these short fragments as templates, random primers, and SuperScript double-stranded cDNA synthesis kit (Invitrogen, Guangzhou, China), double-stranded cDNA was synthesized. The ligated fragments were then generated by a series of reaction processes that included purification of PCR products, end repair, dA-tailing, and ligation of Illumina adapters. After agarose gel electrophoresis, suitable fragments were selected for PCR amplification. An Illumina HiSeq^™^ 2000 sequencing platform was employed to sequence the cDNA library (BerryGenomics, Beijing, China).

### 4. Data filtering and *de novo* assembly

We filtered the raw data to generate clean data via a process that included the removal of adapter sequences, reads with ambiguous sequences “N”, and low-quality sequences (the percentage of low quality bases of quality value _5 was >50% in a read). After obtaining clean data, *de novo* transcriptome assembly was conducted with the short reads assembly program in the Trinity software using default parameters [35, 36]. Only assembled transcripts with lengths of >300 bp were included in subsequent analyses.

### 5. Functional annotation

To annotate the assembled unigenes, all unigenes were analyzed by using the EMBOSS software [37] package to generate putative protein sequences. For the putative protein sequences, we performed the BLASTx search against the NCBI Nr (http://www.ncbi.nlm.nih.gov/genbank/), Swiss-Prot (http://www.uniprot.org/), and KEGG pathway (http://www.genome.jp/kegg/pathway.html) databases, with an E-value cut-off of 1E-5. To further analyze the annotation results, Blast2GO [38] was conducted to obtain the GO functional classification of the unigenes according to molecular function, biological process, and cellular component ontologies (http://www.geneontology.org/). All assembled unigenes were also aligned to the KOG database (http://genome.jgi.doe.gov/) to predict and classify possible functions. The KEGG annotation was performed using the software, KOBAS v2.0 [39].

### 6. SSR mining and primer design

The MIcroSAtellite (MISA, http://pgrc.ipk-gatersleben.de/misa/) was utilized to identify SSR motifs. We screened for motifs with mono-six nucleotides in size and a minimum of 5 contiguous repeat units. Based on MISA results, primer pairs were designed using the software, Primer3 v2.23 [40] with default settings, and the size of the PCR products ranged from 100 bp to 300 bp.

### 7. Survey of SSR polymorphisms

Thirty-five individual buffaloes from 7 breeds in China (S2 Table) were selected for screening SSR polymorphisms. The genomic DNA was extracted from each buffalo blood tissue sample by using the TIANamp Blood DNA Kit (Tiangen Biotech (Beijing) Co., Ltd., Beijing, China), following the manufacturer’s specifications. The DNA concentration was calculated using standard protocols. PCR was performed in 20.0 μL reaction mixtures containing 1.0 μL of the DNA template (10 ng), 1.0 μL of the primer mix (10 μM of each), 10.0 μL of the premixed rTaq solution, and 8.0 μL of ddH_2_O. PCR was conducted in an ABI PCR machine using the following conditions: 3 min at 95°C, followed by 35 cycles of 30 s at 95°C, 30 s at 58°C–60°C, and 30 s at 72°C, and a final extension of 8 min at 72°C. After PCR amplification, the size of each amplified product with 10.0 μL volume was estimated using the LabChip GX instrument (PerkinElmer, USA).

Seven buffalo breeds were selected to validate the amplification and polymorphism of 115 random SSR markers. The values of the observed number of alleles (*N*_*A*_), expected heterozygosity (*H*_*E*_), observed heterozygosity (*H*_*O*_), and polymorphism information content (*PIC*) per SSR locus were calculated using the software, PowerMarker, version 3.25 [41]. An UPGMA hierarchical clustering was performed based on the matrix of genetic similarity estimates, following the procedures of the PowerMarker software.

## Results

### 1. Sequencing and *de novo* assembly of swamp buffalo transcriptome

**Fig 1:**
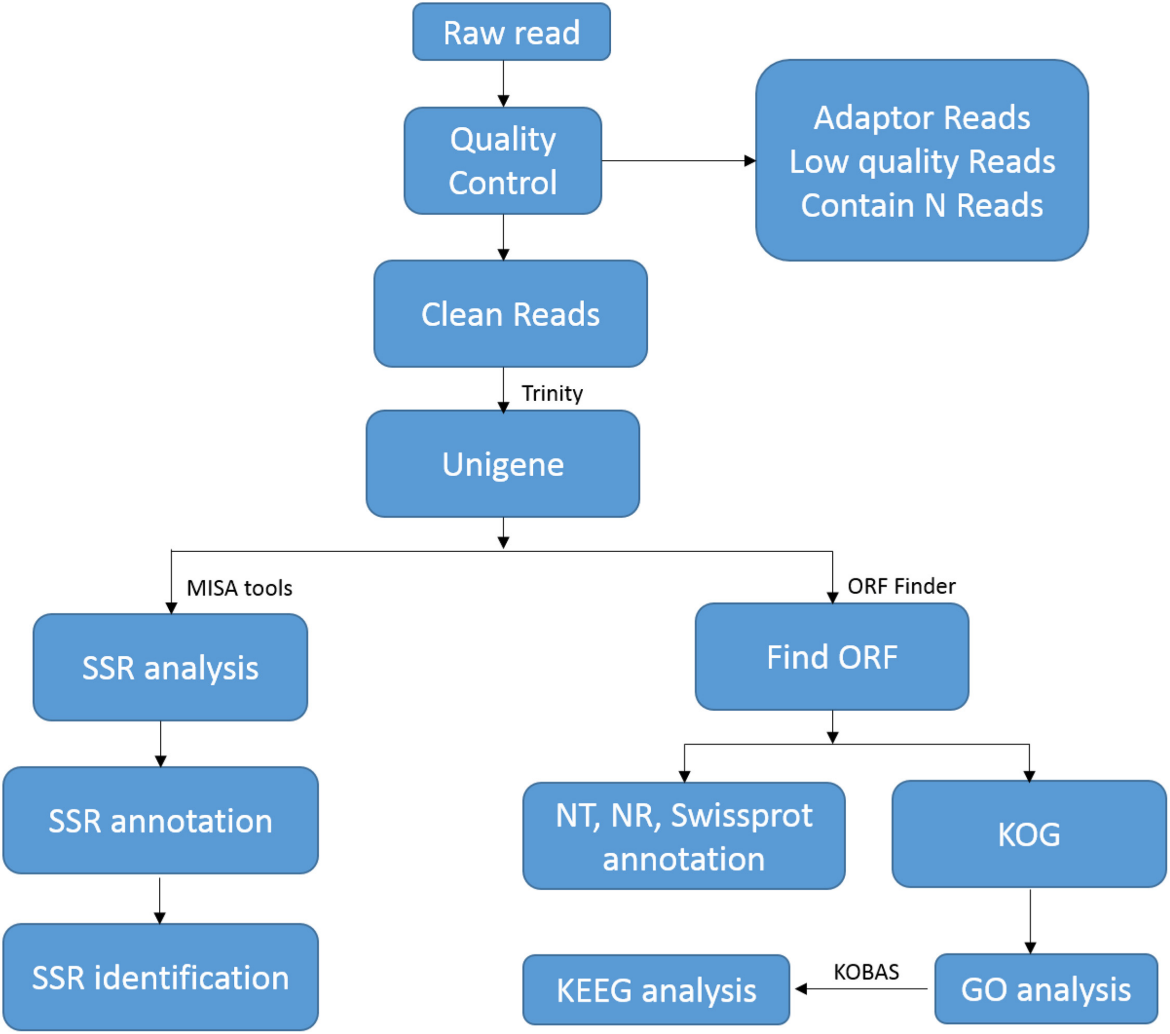
Flowchart of *de novo* assemble in swamp buffalo transcriptome and SSR discovery.

To obtain a comprehensive overview of the swamp buffalo (*B*. *bubalis*) transcriptome, we performed transcriptome sequencing of pooled RNA samples from 11 different tissues on the Illumina Hiseq 2000 platform. The main steps and bioinformatics tools used for data analysis are shown in Fig 1. We obtained a total of 54,109,173 raw reads, which after removal of redundant reads, trimming of adaptors and filtering for low-quality sequences resulted in 52,979,055 high-quality clean reads with 10,595,811,000 bp of sequence data (Table 1). The results of FastQC v0.11.3 analysis showed that the Q20 percentage and GC percentage were 97.91% and 49.92%, respectively. Using the Trinity software (Release-20140717), *de novo* assembly was performed, which yielded 86,017 unigenes with a mean length of 972.41 bp and an N50 of 1,505 bp, representing a total of 83.65 Mb of genomic sequence. The present study was named the Transcriptome Shotgun Assembly Project, and the 86,017 unigenes identified in swamp buffalo were deposited in GenBank under accession number GDJS00000000.1 (http://www.ncbi.nlm.nih.gov/nuccore/954037469?log$=activity). Of these deposited unigenes, 47,929 (55.72%) unigenes were >500 bp in size, 22,279 unigenes (25.90%) were >1,000 bp in size, and 9,969 (11.59%) unigenes were >2,000 bp long. According to a simple principle: the longest one was extracted when the unigenes had multiple open reading frames (ORFs), 76,703 (89.17%) unigenes with ORFs were generated using the software, ORF Finder (EMBOSS:6.3.1), which indicated that 8,740 unigenes had complete ORFs, with an average GC content of 44.5% (data not shown).

**Table 1:** Summary of results of sequence analysis.

**Data generation and filtering**	
Raw reads	54,109,173
Clean reads	52,979,055
Q20 percentage (%)	97.04
GC content (%)	49.92
**Assembly statistics**	
300–500 (bp)	38,088 (44.28%)
500–800 (bp)	19,941 (23.18%)
800–1,000 (bp)	5,713 (6.64%)
1,000–1,500 (bp)	7,916 (9.20%)
1,500–2,000 (bp)	4,393 (5.11%)
>2,000 (bp)	9,970 (11.59%)
Unigenes	86,017
Total length (bp)	83,647,650
N50 length (bp)	1,505
Mean length (bp)	972.41

### 2. Functional annotation

**Fig 2:**
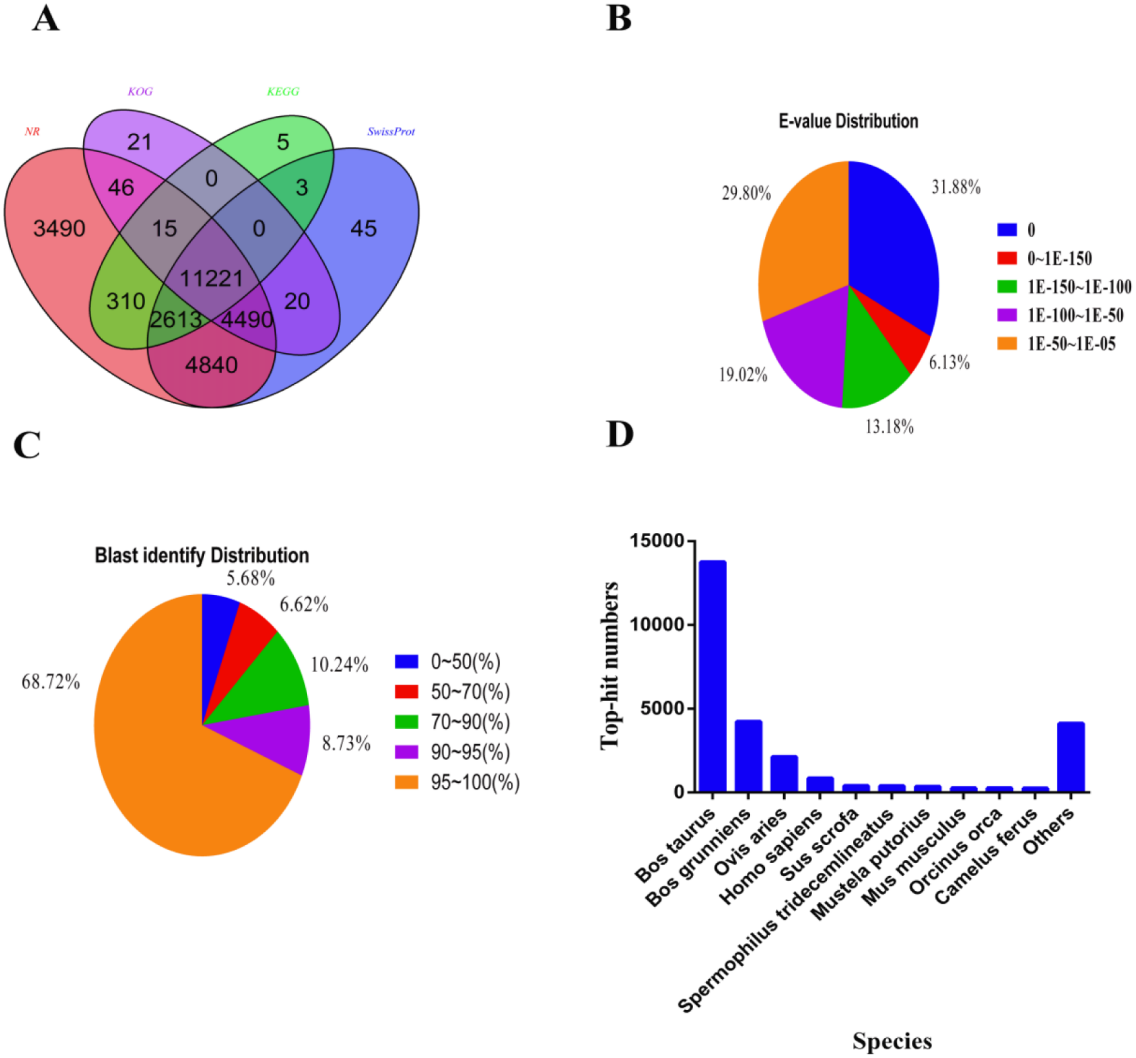
Characteristics of the results of homology search for swamp buffalo unigenes. (**A**) Venn diagram of BLAST hits for unigenes against protein databases (E-value ≤1.0e-05). Numbers in the circles indicate the number of unigenes annotated by single or multiple databases, (**B**) E-value distribution of BLAST hits for each unique sequence (E-value ≤1.0e-05). (**C**) Identity distribution of BLAST hits for each sequence. (**D**) Species distribution of the top BLAST hits for the assembled unigenes (E-value ≤1.0e-05).

The assembled unigenes were predicted by using the BLASTx [42] program against the NCBI non-redundant (Nr) and Swiss-Prot protein databases, with an E-value threshold of 1E-5. Among the 86,017 unigenes, 27,025 (31.41%) and 23,232 (27.00%) unigenes showed significant similarity to known proteins in the Nr and Swiss-Prot databases, respectively. Furthermore, 15,813 and 14,167 unigenes could be annotated according to the EuKaryotic Ortholog Groups (KOG) and Kyoto Encyclopedia of Genes and Genomes (KEGG) pathways [43], respectively (Fig 2A). The E-value distribution of the hits showed that 70.20% of the unigenes had significant homology (< 1E-50) to entries in the Nr database, and nearly 87.69% of the sequences showed >70.00% similarity (Fig 2B and 2C). The 86,017 unigenes were annotated to 10 top-hit species, with *Bos taurus* and *B*. *grunniens* accounting for 66.52% of the annotated unigenes (Fig 2D). These results revealed that our transcriptome data on the swamp buffalo was successfully annotated.

**Fig 3:**
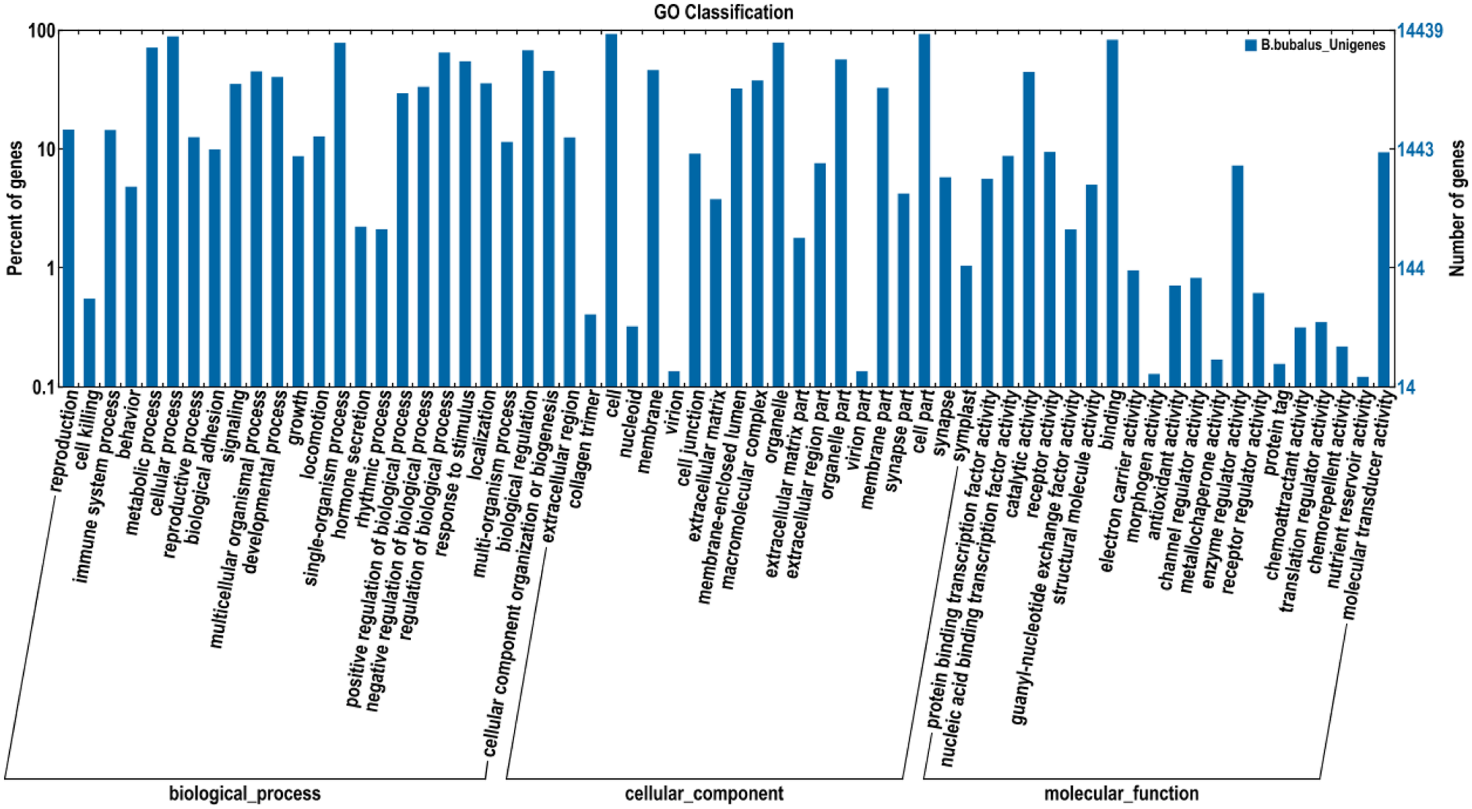
Gene ontology (GO) classification of assembled unigenes. A total of 14,439 unigenes with significant similarity in NR protein databases were assigned to GO classifications.

Based on the results of Nr annotation, 14,439 unigenes were assigned to 64 functional groups in Gene Ontology (GO) [44]. Fig 3 shows that 112,386 (53.04%) unigenes comprised the largest category, namely, ‘biological process’, followed by ‘cellular component’ (73,975; 34.91%) and molecular function (25,535; 12.05%). The GO terms ‘cellular process’ (12,727; 11.32%) and ‘single-organism process’ (11,225; 9.99%), ‘cell’ (13,314; 18.00%), and ‘cell part’ (13,313; 18.00%), and ‘binding’ (11,917; 46.67%), and ‘catalytic activity’ (6,379; 24.98%) were the first and second largest groups among the three main categories (‘biological process’, ‘cellular component’, and ‘molecular function’), respectively. However, a few unigenes were assigned to ‘virion’ (GO: 0019012), ‘virion part’ (GO: 0044423), ‘morphogen activity’ (GO: 0016015), and ‘nutrient reservoir activity’ (GO: 0045735).

**Fig 4:**
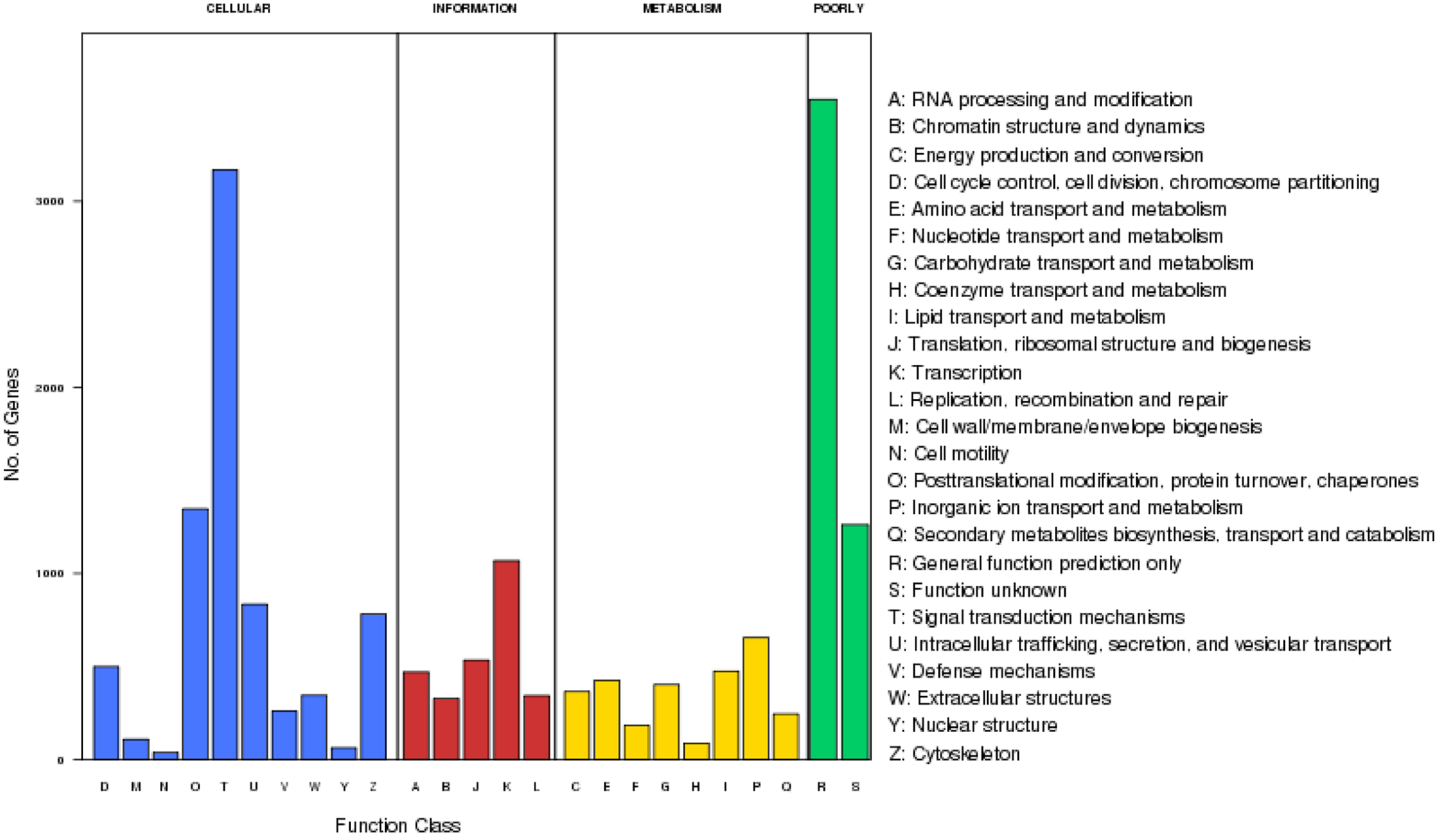
EuKaryotic orthologous group (KOG) classification. Approximately 15,813 of the 86,017 unigenes with NR hits were grouped into 25 KOG classifications.

In addition, all unigenes were subjected to a search against the KOG database for functional prediction and classification. A total of 15,813 unigenes showing Nr hits in the KOG database were functionally classified into 25 molecular families, including four orthology clusters (Fig 4). The orthology cluster described as ‘cellular processes and signaling’ predominated, which accounted for 41.75% of the annotations, followed by ‘metabolism’ (2,839; 15.92%) and ‘information storage and processing’ (2,742; 15.37%); whereas, another clustering was poorly characterized, which included ‘general prediction only’ and ‘function unknown’, which accounted for 26.96% of the annotations.

### 3. Functional classification using the KEGG pathway

**Fig 5:**
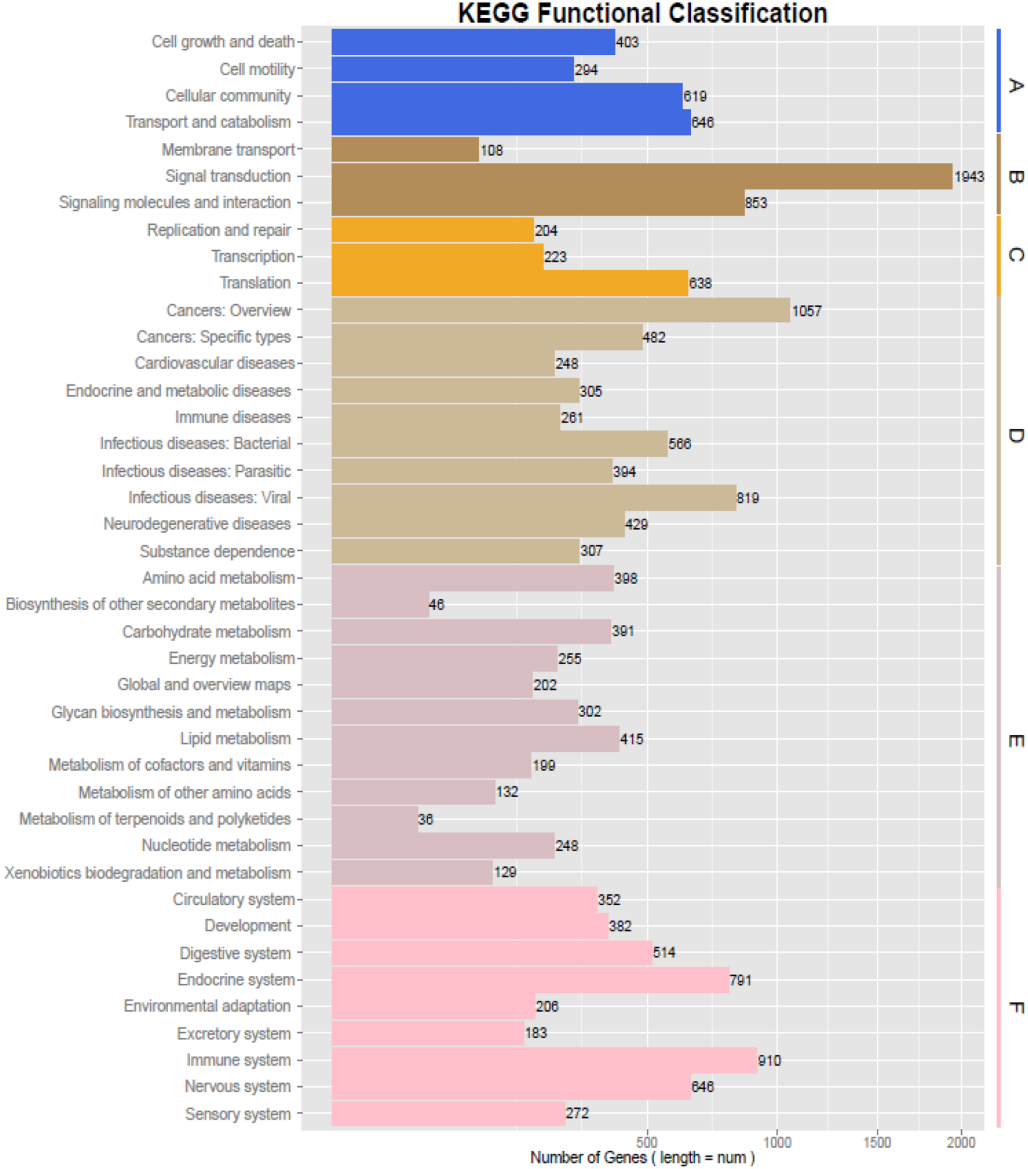
Pathway assignment based on the Kyoto Encyclopedia of Genes and Genomes (KEGG). (**A**) Classification based on cellular processes categories, (**B**) Classification based on environmental information processing categories, (**C**) Classification based on genetic information processing categories, (**D**) Classification based on human diseases categories, (**E**) Classification based on metabolism categories, and (**F**) Classification based on organismal systems categories.

All the assembled unigenes were subjected to KEGG pathway enrichment analysis. A total of 14,167 unigenes (16.47%) could be annotated and assigned to 5 main categories, which included 331 KEGG pathways (Fig 5, S1 Table). Among the five main categories, the largest category was ‘human diseases’, which contained 4,868 KEGG-annotated unigenes (26.52%), followed by ‘organismal systems’ (4,256; 23.19%), ‘environmental information processing’ (2,904; 15.82%), ‘metabolism’ (2,753; 15.00%), ‘cellular processes’ (1,962; 10.69%), and ‘genetic information processing’ (1,613; 8.79%). S1 Table shows that the KEGG human diseases contained 10 subcategories, which included Cancers: overview, Cancers: specific types, cardiovascular diseases, Endocrine and metabolic diseases, Immune diseases, Infectious diseases: Bacterial, Infectious diseases: Parasitic, Infectious diseases: Viral, Neurodegenerative diseases, and Substance dependence. Furthermore, 415 unigenes were assigned to the subcategory of Lipid metabolism. Among these, 34, 20, and 21 unigenes mapped to the subcategories of Linoleic acid metabolism, Alpha-linolenic acid metabolism, and Biosynthesis of unsaturated fatty acids, respectively.

### 4. Development and characterization of SSR markers

SSRs are useful molecular markers for genetic research and comparative genome analysis. To develop SSR markers in swamp buffalo, all assembled unigenes generated in the present study were used to mine potential microsatellites, which were defined as mono- to hexanucleotide SSRs that consisted of a minimum of five repeats. Table 2 presents the 18,446 SSRs that were detected in 17,401 unigenes, of which 2,939 unigenes contained more than one SSR, and 932 SSRs exhibited compound formation. The number of potential SSRs per unigene varied from 1 to 6, with an average of 1.06.

**Table 2:** Summary of SSR mining results.

Search item	Number
Total number of sequences examined	86,017
Total size of examined sequences (bp)	83,647,650
Total number of identified SSRs	18,446
Number of unigenes containing SSRs	17,401
Number of unigenes containing more than 1 SSR	2,939
Number of SSRs present in compound formation	932
Number of mononucleotides	1,0557
Number of dinucleotides	3,580
Number of trinucleotides	2,162
Number of tetranucleotides	149
Number of pentanucleotides	14
Number of hexanucleotides	7

To further assess the mining quality of SSRs in swamp buffalo, we divided the SSRs into three groups based on the repeat motif classification criteria proposed by Weber [45] (Table 3). For the perfect repeat motifs (SSRs ≥15 bp in length), mono-, tri-, and dinucleotide motifs were placed as top three hits, with distribution frequencies of 38.53%, 36.08% and 22.56%, respectively, whereas the other motif types only accounted for 2.84% of the repeat motifs. Under the imperfect SSR category, 10,476 SSRs was detected, which included mono- (8,248; 78.73%) and dinucleotide (2,228; 21.27%) SSR units, and was ranked after the perfect repeat motifs. For the compound SSR category, all motifs belonged to the perfect type, including the mono-mono-, mono-di-, mono-tri-, mono-tetra-, di-mono-, di-di-, di-tri-, di-tetra-, tri-mono-, tri-di-, tri-tri-, tetra-tetra-, and hexa-trinucleotide types. The mono-mono-, di-di-, and tri-trinucleotide types were the most abundant, representing more than 77.04% of the 932 SSRs.

**Table 3:** Repeat motif type distribution in SSRs ≥ 15 bp in length.

Repeat motif type	SSRs ≥ 15 bp in length	
	Number	Frequency (%)
Perfect		
Mono-	2,309	38.53
Di-	1,352	22.56
Tri-	2,162	36.08
Tetra-	149	2.49
Penta-	14	0.23
Hexa-	7	0.12
Total	5,993	100.00
Imperfect		
Mono-	8,248	78.73
Di-	2,228	21.27
Total	10,476	100.00
Compound		
Perfect		
Mono-mono-	440	47.21
Mono-di-	77	8.27
Mono-tri-	21	2.25
Mono-tetra-	6	0.64
Di-mono-	78	8.37
Di-di-	218	23.39
Di-tri-	5	0.54
Di-tetra-	3	0.32
Tri-mono-	17	1.82
Tri-di-	5	0.54
Tri-tri-	60	6.44
Tetra-tetra-	1	0.11
Hexa-tri-	1	0.11
Total	932	100.00
Total	17,401	

**Fig 6:**
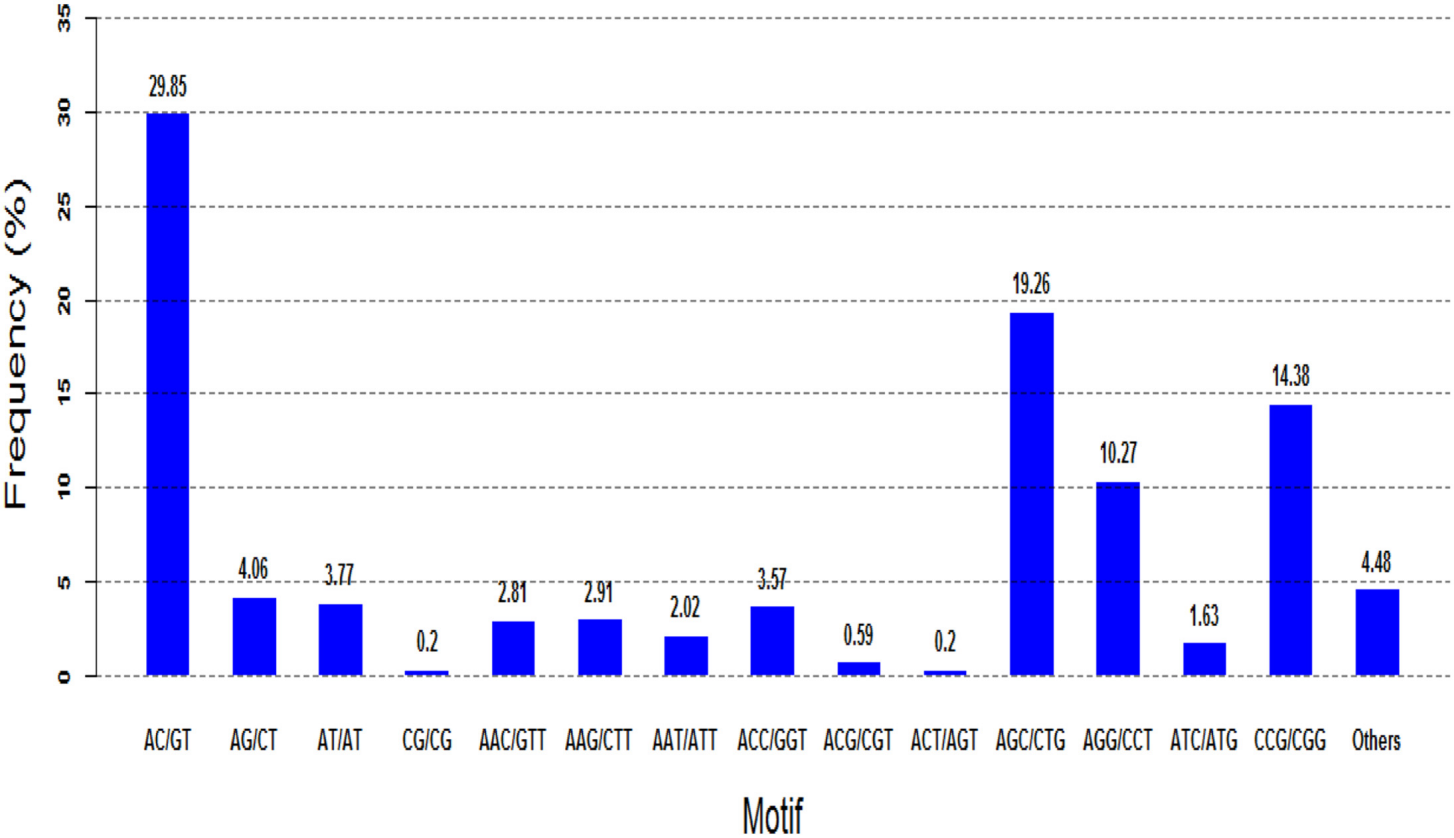
Frequency of classified repeat types of SSRs. The most abundant dinucleotide and trinucleotide motifs were AC/GT and AGC/CTG.

The frequency distribution of the perfect SSRs was also analyzed in the present study, with the mononucleotide type excluded. The most abundant motif detected in the SSRs was the AC/GT motif (29.85%), followed by the motifs AGC/CTG (19.26%), CCG/CGG (14.38%), and AGG/CCT (10.27%). The remaining types of motif accounted for 26.24% of the repeat motifs (Fig 6).

### 5. Identification of polymorphic markers

**Fig 7:**
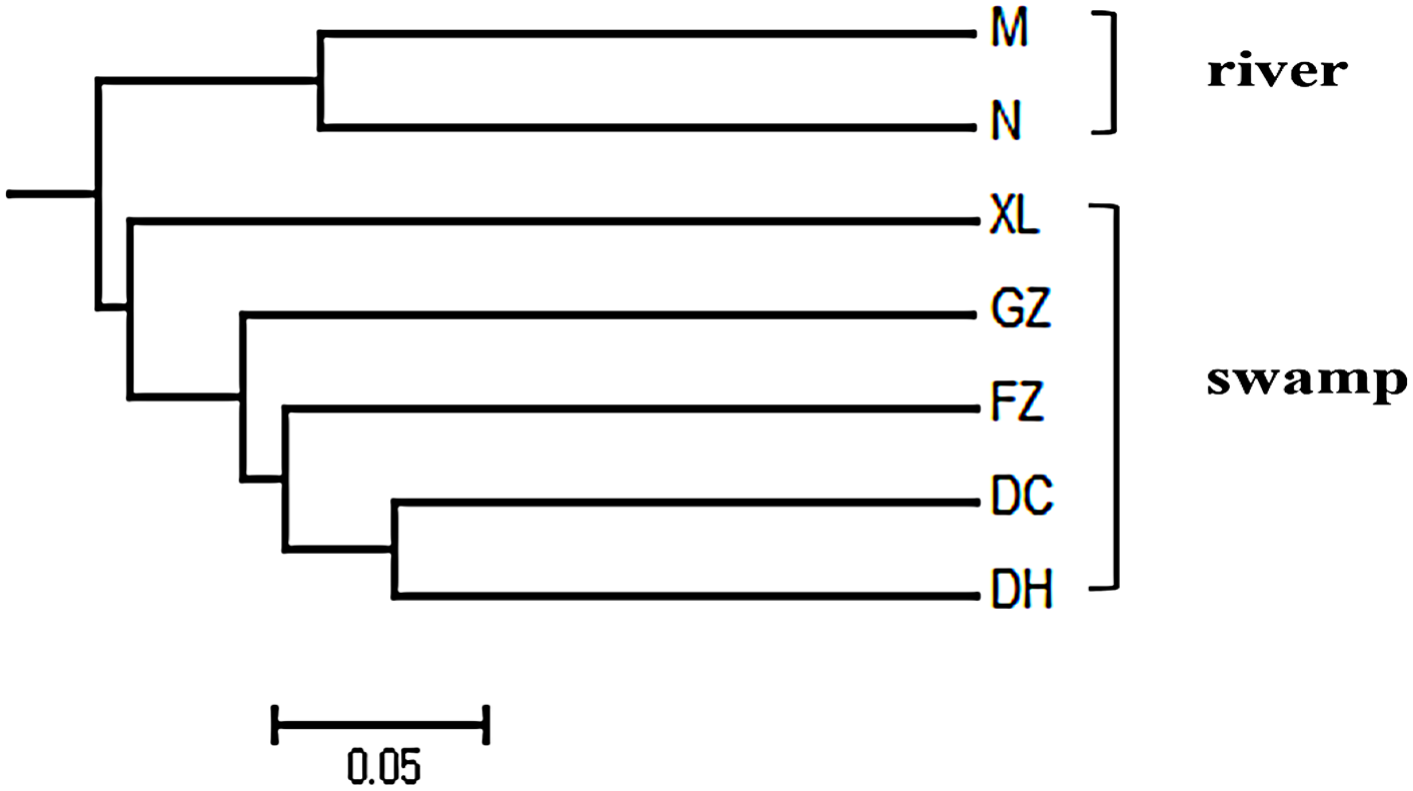
UPGMA dendrogram of the genetic relationships among seven buffalo breeds. The dendrogram was generated using the Nei similarity coefficient based on 69 polymorphic primer pairs.

One hundred and fifteen SSRs were randomly selected to estimate the genetic diversity of 7 buffalo breeds (S3 Table). We successfully amplified PCR products using 110 primer pairs, and 69 primer pairs exhibited polymorphisms among the 7 breeds. Of the 69 working primer pairs, 52 PCR products showed specific amplification with the expected sizes, whereas the other 17 PCR products were larger than the expected sizes, suggesting that the amplified regions likely contained introns. Table 4 shows the average values of the *N*_*A*_, *H*_*E*_, and *H*_*O*_ in the 69 SSRs, which were 9.00, 0.74, and 0.18, respectively. The *PIC* values ranged from 0.33 to 0.91, with an average value of 0.70, suggesting that those highly polymorphic markers could be used to investigate genetic diversity in buffalo. The unweighted pair group method with arithmetic mean (UPGMA) cluster analysis divided 7 breeds into two groups, one representing the river buffalo types (Murrah and Nili-Ravi), whereas the other comprised all the indigenous breeds (5 swamp types) (Fig 7). For the swamp buffalo group, the DC swamp buffalo was closely related to the DH swamp buffalo.

**Table 4:** Characterization of 69 SSRs in the swamp buffalo.

Unigene	Size range (bp)	*N*_*A*_	*H*_*E*_	*H*_*O*_	*PIC*
c100962_g1	179–275	16	0.83	0.69	0.81
c101428_g1	138–220	16	0.89	0.25	0.88
c95720_g2	231–273	8	0.70	0.00	0.66
c86603_g2	223–257	5	0.57	0.04	0.49
c96868_g2	266–290	7	0.70	0.03	0.64
c98098_g9	176–185	7	0.67	0.00	0.62
c97555_g6	174–212	12	0.81	0.03	0.80
c97496_g2	238–241	5	0.72	0.03	0.66
c97325_g1	183–258	5	0.75	0.00	0.71
c90560_g1	272–274	2	0.50	0.00	0.37
c95590_g1	264–269	6	0.66	0.00	0.61
c29117_g1	249–284	11	0.83	0.46	0.81
c3537_g1	235–252	8	0.75	0.10	0.72
c90817_g1	142–147	5	0.71	0.00	0.66
c95357_g1	211–227	10	0.82	0.43	0.80
c95815_g1	296–299	4	0.58	0.00	0.49
c90328_g2	250–279	13	0.87	0.21	0.86
c90309_g3	118–206	7	0.67	0.16	0.61
c90620_g1	255–276	11	0.81	0.43	0.79
c85589_g3	232–272	10	0.82	0.42	0.81
c97420_g2	194–225	15	0.91	0.00	0.90
c30689_g1	205–210	6	0.75	0.00	0.71
c90393_g2	160–256	10	0.83	0.10	0.82
c90478_g1	410–465	15	0.88	0.86	0.87
c90599_g1	208–223	6	0.62	0.11	0.56
c95402_g8	183–197	5	0.66	0.11	0.61
c97823_g8	100–105	6	0.81	0.00	0.78
c56032	144–180	7	0.79	0.39	0.76
c63011_g1	364–375	12	0.88	0.00	0.87
c95392_g3	226–228	3	0.65	0.00	0.58
c95505_g1	269–273	5	0.75	0.00	0.71
c96324_g1	246–274	9	0.74	0.50	0.71
c95394_g1	200–285	6	0.76	0.03	0.72
c99660_g1	142–200	8	0.68	0.45	0.65
c95544_g1	235–237	3	0.44	0.00	0.39
c43761_g2	149–269	16	0.74	0.37	0.71
c97820_g4	236–240	5	0.75	0.00	0.72
c97498_g3	164–168	4	0.59	0.00	0.52
c94999_g1	173–232	10	0.73	0.18	0.69
c96337_g7	246–248	3	0.49	0.00	0.39
c96483_g1	181–207	9	0.76	0.29	0.72
c91113_g1	194–274	14	0.83	0.24	0.81
c90599_g1	238–240	3	0.37	0.00	0.33
c90878_g3	180–272	16	0.89	0.43	0.88
c650_g1	270–274	5	0.73	0.00	0.68
c95889_g10	176–216	13	0.89	0.09	0.88
c90552_g3	127–175	19	0.92	0.63	0.91
c90374_g1	370–390	8	0.60	0.15	0.53
c94121_g3	212–235	8	0.73	0.18	0.68
c90300_g1	157–160	4	0.62	0.00	0.54
c98127_g11	175–204	12	0.85	0.71	0.84
c95978_g1	242–280	11	0.84	0.29	0.82
c92172_g3	201–271	11	0.84	0.03	0.82
c99615_g1	262–277	10	0.79	0.13	0.76
c92254_g5	186–294	13	0.86	0.07	0.85
c29773_g1	252–293	19	0.91	0.57	0.91
c45669_g1	190–194	5	0.78	0.00	0.75
c96108_g12	164–230	17	0.86	0.62	0.84
c91434_g2	171–284	9	0.79	0.51	0.77
c90483_g5	269–290	13	0.80	0.37	0.78
c96873_g3	184–188	5	0.74	0.00	0.70
c97681_g7	158–275	6	0.46	0.03	0.43
c28863_g1	170–226	7	0.69	0.09	0.64
c97995_g3	162–283	7	0.60	0.03	0.56
c98267_g4	160–231	6	0.61	0.09	0.54
c98164_g4	134–200	11	0.67	0.11	0.65
c78294_g2	181–184	4	0.43	0.00	0.41
c90756_g3	154–246	15	0.91	0.03	0.91
c88071_g3	178–264	12	0.88	0.06	0.86
Mean		9	0.74	0.18	0.70

Note: *N*_*A*_, number of alleles; *H*_*E*_, expected heterozygosity; *H*_*O*_, observed heterozygosity; *PIC*, polymorphic information content

## Discussion

The Chinese swamp buffaloes have been divided into 14 local types and many populations based mainly on regional distribution [46]. Not only are they draught animals, but they also have a tremendous economic importance as dairy and meat in many highly populated countries [47, 48]. Despite their utility, studies on genomics as a tool for marker assisted cross-breeding techniques are still lacking in this species because of strategies which were relatively costly, time consuming and labor intensive [49]. The high demand for low-cost sequencing has led to the development of high-throughput technologies such as next-generation sequencing [50]. Transcriptome sequencing is one such powerful and cost-effective tool in generating large-scale transcriptome data that may be used in developing molecular markers and in identifying novel genes in model [35, 51] and non-model [52, 53] organisms. To our knowledge, the present study is the first attempt to perform *de novo* assembly and to conduct a comprehensive characterization of the comprehensive transcripts of swamp buffalo. We obtained a total of 52,979,055 high-quality reads with 97.04% Q20 bases using Illumina paired-end sequencing, and *de novo* assembly yielded 86,017 unigenes, which might be useful for further research into functional genomics in the swamp buffalo. The average length of the assembled unigenes with an N50 of 1,505 bp was 972.41 bp, which was longer than the results of previous studies [54–56], suggesting that our transcriptome sequencing data was successfully assembled. The 49.92% GC-content of the swamp buffalo transcriptome was higher than the genome-wide average GC-content of the river buffalo draft genome (42.20%) and those of other animals (41.80%–42.30%) [57–60], which might be attributable to the unique tissue-specific transcripts and experimental designs [61, 62]. These results are indicative that the transcripts generated from the swamp buffalo were of high quality and may thus be utilized in future studies on gene cloning, molecular genetics, and transgenesis of the swamp buffalo.

To predict and analyze the biological function of assembled transcripts at the whole-transcriptome level, a sequence similarity search was performed against various protein databases, which included Nr, Swiss-Prot, GO, KOG, and KEGG. Most of the assembled unigenes (62,337; 72.47%) showed matches with known proteins in public databases, indicating that 27.53% of the unigenes may represent novel genes whose function has not yet been identified. In particular, most of unigenes were annotated to the *B*. *taurus* and *B*. *grunniens* (first and second hits) against the Nr database, probably because: (1) it confirmed that the swamp buffalo is closely related to *B*. *taurus* and *B*. *grunniens*; (2) The genomes of both *B*. *taurus* and *B*. *grunniens* have earlier been completely sequenced [63, 64]. We mapped 23.16% of the annotated unigenes to the KOG database and 25.37% to the GO terms, which indicated that our transcriptome data represented a broad diversity of transcripts in swamp buffalo. Similar results were also reported in other species, such as sheep [65], fish [66], horse [67], rubber tree bark [68], the Tibetan leguminous shrub *Sophora moorcroftiana* [69], and the Jerusalem artichoke [70]. On the other hand, around 26.96% of the annotated unigenes were poorly characterized to orthologous clusters and thus were described as ‘general prediction only’ and ‘function unknown’; this occurrence may be due to the absence of a reference genome for the swamp buffalo. In addition, we also predicted a total of 14,167 unigenes that mapped to 331 KEGG pathways. Moreover, 70.00% of the top 10 hit pathways were involved in signal transduction, whereas the others were related to pathways involving cancer, proteoglycans in cancer, and HTLV-I infection (S1 Table). Notably, some unigenes predicted by KEGG pathways were associated with linoleic acid metabolism, alpha-linolenic acid metabolism, and biosynthesis of unsaturated fatty acids, implying that swamp buffalo milk is very rich in unsaturated fatty acids and has important economic value and health benefits. These results indicated that the predicated pathways, together with gene annotation, may be utilized in future investigations on gene function, which in turn also confirms that *de novo* transcriptome sequencing is an efficient method for transcriptome characterization and gene discovery in the swamp buffalo.

SSRs that are widely distributed in a genome are important tools for assessing genetic diversity, genetic map construction, comparative genomics, and marker-assisted selection breeding. To our knowledge, no previous study has identified SSR markers in the swamp buffalo. The transcriptome data is an excellent source for SSR mining and has been utilized in various species [71–74]. In the present study, we identified a total of 17,401 SSRs based on the unigene data of swamp buffalo and approximately 39.80% of identified SSRs were the perfect repeat motif type. When mononucleotide repeats were excluded, 48.61% of the 4,616 SSRs were determined to be trinucleotide repeats, followed by dinucleotide repeats (35.88%) and tetranucleotide repeats (3.25%), as well as pentanucleotide repeats and hexanucleotide repeats, which accounted for 0.48% of the motifs. The most abundant dinucleotide and trinucleotide motifs were AC/GT and AGC/CTG (Fig 5), which was in agreement with the findings of previous reports on other animal species [55, 75, 76], but different from those of plants [68, 77]. Of the 115 primer pairs randomly selected for PCR validation, 110 (95.65%) produced clear bands, and 69 (60.00%) exhibited polymorphisms. The high PCR rate of SSR markers in the swamp buffalo was similar to that obtained in other species [71, 78], but higher than that reported in a study conducted by Yan [75]. UPGMA dendrogram analysis revealed that the two river buffalo populations clustered together whereas the five swamp buffalo populations were clustered separately, which correlated with the geographic origin of the genotypes. The findings of UPGMA analysis was similar to that observed in previous studies [13, 79, 80]. In sum, the 17,401 potential SSRs identified in the present study provide a useful resource for future marker assisted breeding programs in the swamp buffalo.

## Conclusions

In the present study, Illumina paired-end sequencing was performed, followed by *de novo* assembly and characterization of the transcriptome of the swamp buffalo. Our study generated a total of 54,109,173 raw reads, which consisted of 86,017 unigenes, of which 62,337 unigenes were annotated to the four public databases (Nr, Swiss-Prot, KOG, and KEGG), which in turn identified 17,401 SSRs as putative molecular markers. These findings may serve as a valuable resource for genetic and genomic studies on the buffalo.

## Supporting Information

S1 Table Summary of KEGG classification of assembled unigenes, (XLSX)

S2 Table Characteristics of seven buffalo breeds for SSR validation, (DOCX)

S3 Table Primers information for SSRs in swamp buffalo, (XLSX)
