# Supplementary material for: De Novo Transcriptome Assembly of the Chinese Swamp Buffalo by RNA Sequencing and SSR Marker Discovery
Source: PLoS One. 2016 Jan 14;11(1):e0147132. doi: 10.1371/journal.pone.0147132 (PMC4713091; doi:10.1371/journal.pone.0147132)
Supplement: S2 Table — (DOCX) [file pone.0147132.s002.docx]

**S2 Table. Characteristics of seven buffalo breeds for SSR validation**

| Population | Code | Sources | N | Domestication | Types of buffalo |
| --- | --- | --- | --- | --- | --- |
| Dechang | DC | Sichuan, China | 5 | Indigenous | Swamp |
| Dehong | DH | Yunnan, China | 5 | Indigenous | Swamp |
| Fuzhong | FZ | Guangxi, China | 5 | Indigenous | Swamp |
| Xilin | XL | Guangxi, China | 5 | Indigenous | Swamp |
| Guizhou | GZ | Guizhou, China | 5 | Indigenous | Swamp |
| Murrah | M | India | 5 | Exotic | River |
| Nili-ravi | N | Pakistan | 5 | Exotic | River |

Note: N=sample size
